# A Comparison of HbA1c and Fasting Blood Sugar Tests in General Population

**Published:** 2010

**Authors:** Zahra Ghazanfari, Ali Akbar Haghdoost, Sakineh Mohammad Alizadeh, Jamileh Atapour, Farzaneh Zolala

**Affiliations:** 1 Kerman University of Medical Science, Physiology Research Centre, Jahad street, Kerman, Iran; 2 Kerman University of Medical Science, Faculty of Health, Haftbagh-Alavi Highway, Kerman, Iran

**Keywords:** HbA1c, Blood glucose, Diabetics

## Abstract

**Objectives:**

Early diagnosis of diabetes is crucially important in reduction of the complications. Although HbA1c is an accurate marker for the prediction of complications, less information is available about its accuracy in diagnosis of diabetes. In this study, the association between HbA1c and FBS was assessed through a cross-sectional population-based study.

**Methods:**

A random sample of population in Kerman city was selected. The total number was 604 people. Their HbA1c and fasting blood sugar (FBS) were tested. The association between HbA1c and FBS and also their sensitivity, specificity and predictive values in detection of abnormal values of each other were determined.

**Results:**

The association of HbA1c with FBS was relatively strong particularly in diabetic subjects. Generally, FBS was a more accurate predictor for HbA1c compared with HbA1c as a predictor of FBS. Although the optimum cutoff point of HbA1c was >6.15%, its precision was comparable with the conventional cutoff point of >6%.

**Conclusions:**

In conclusion, FBS sounds more reliable to separate diabetic from non-diabetic subjects than HbA1c. In case of being interested in using HbA1c in screening, the conventional cutoff points of 6% is an acceptable threshold for discrimination of diabetics from non-diabetics.

## INTRODUCTION

The burden of type 2 diabetes has a rising trend in the world. The worldwide prevalence of diabetes among general population was estimated at 150 millions in 1995, and this is projected to increase to 300 millions by 2025.^1^ Developing countries such as most of the Middle Eastern countries are experiencing an accelerated rate in this issue.^2^ Iran is a large Middle East country with around 74 million population with different ethnic groups; 68% of population are residents of urban areas. In a systematic review of descriptive studies in Iran, the prevalence of diabetes was estimated around 24% in those aged over 40, with a higher prevalence in females.^3^ The literature advocate the importance of early diagnosis in order to reduce diabetes complications.^4^ It is estimated that about one third of people with type 2 diabetes might be undiagnosed until the complications are developed.^5^ Therefore, establishing efficient screening programs to detect people with undiagnosed diabetes is important. A screening test is discussed to be effective when a series of conditions are met. These criteria are categorized in 10 groups such as a considerable number of people are being affected, screening test could lead to early diagnose and also existing effective treatments.^6^ In order to detect diabetics, fasting blood glucose (FBS) is suggested as the best and the most common test with the cutoff point >126 mg/dl.^2^ However, there are some issues about using FBS such as keeping the clients fast for about 8 hours and not being applicable in the afternoon. Besides, in centralized screening when laboratory facilities are available, HbA1c test, which is the percentage of glycated hemoglobin is recommended to measure the incidence or prevalence.^2^ Apart from the efficacy of HbA1c in detection of diabetes, it is an important marker to assess the microvascular complications and plasma glucose.^7^ The relationship between HbA1c and blood glucose is documented in the literature denoting a straight relationship.^8^–^10^ However, this relationship has not been confirmed by others.^11^ There is a controversy about the performance of HbA1c in case finding. It has been argued that due to problems in standardization and variations in styles of HbA1c test, it is not recommended as a routine test for screening of diabetes.^12^ In addition, other factors such as abnormal hemoglobin, anemia and some drugs may affect the results of HbA1c test.^13^ Also, demographic factors such as race and gender are other effective factors.^14^,^15^ Saudek and his colleagues compared FBS and HbA1c as screening tests. They argued that HbA1c is preferable because it is more time-flexible and informative in long-term conditions. Their criterions have been stabilized in recent years.^16^ However, in an epidemiological study, it has been concluded that FBS is more accurate than HbA1c.^17^ The best cutoff point for defining high HbA1c is another important issue. The Diabetes Control and Complications Trial suggested the value of 6% as HbA1c cut point.^18^ The United Kingdom Prospective Diabetes Study considered 6.2 as the normal level,^19^ while many laboratories consider 4-6 as a normal range.^20^ It seems that in different settings such as screening, diagnosis and prediction of progression of diabetes we need to define different cut off points. For example, It is suggested the value of 6.5% or greater as a diabetes diagnostic criterion and 6% and 4.7 for screening test.^16^,^21^ Inoue and his colleagues used the value of 5.8% for the prediction of progression of diabetes type 2,^22^ and the value <7 as a good predictive of satisfactory blood glucose control in type 1 diabetes.^23^ The main aim of major primary studies carried out in diabetes in Iran was to recognize the range of HbA1c in the diabetics, tracing back the complications of diabetes and diabetes control.^10^,^24^,^25^ A few researches have been carried out to find out the cutoff value of HbA1c in screening; however, they mainly used a selective samples mainly focusing on high risk groups.^26^,^27^ Furthermore, a study has determined the normal range of HbA1c in a sample of non-diabetics.^28^ Based on the above explanation and to fill the gaps, this study aimed to explore the relationship of HbA1c and FBS in general population. In addition, the optimum cutoff point of HbA1c for separation of diabetics and non-diabetics was explored.

## METHODS

This cross-sectional population based study was carried out in Kerman city, which is the center of the largest province in south east of Iran. Considering 80% as the minimum sensitivity and specificity of HbA1c in the prediction of FBS>126mg/dl, precision of 3% and significant level of 95%, sample size of 600 people were computed. A total number of 604 individuals were recruited. The city is divided into 30 postal areas. Participants were recruited through a proportional cluster sampling across the city. In each household, people between 18 and 75 years were informed verbally about the study objectives and were requested for their participation. All participants who were invited to participate in the study, agreed for collaboration. They were given a written formal consent form. Since subjects were requested to attend in our reference laboratory for taking blood samples, those who could not attend or did not consent were excluded. The data were collected through a structural face to face interview and laboratory tests. Data collection form included two main sections, the demographic characteristics and also some questions about history of diabetes and taking medications. FBS was measured once using enzyme method and the cutoff point of 126 mg/dl.^2^ was considered as diagnostic criterion for the diabetes. In order to measure HbA1c, we used Biosystem kit. The cutoff point of 6%, based on the Diabetes Control and Complications Trial,^18^ was considered as diagnostic criterion for diabetes at the beginning. Then, we checked the sensitivity and specificity of other cutoff points using ROC curve to optimize the accuracy of HbA1c in detection of FBS>126 mg/dl. Afterwards, parameters including sensitivity, specificity, predictive values (positive and negative), and the chance of detecting diabetes and the chance of ruling out diabetes before and after test were calculated. Data were analyzed from descriptive and analytical point of view using Stata software, version 10. The Pearson correlation coefficient between HbA1c and FBS was estimated in the whole sample, and in sub-groups (diabetics versus non-diabetics). In order to explore the effects of possible confounder, in the univariate analysis, the association between each variable and the FBS and also HbA1c was checked. Variables with univariate p value <0.1 were entered in multivariate analysis.

## RESULTS

**Figure 1:**
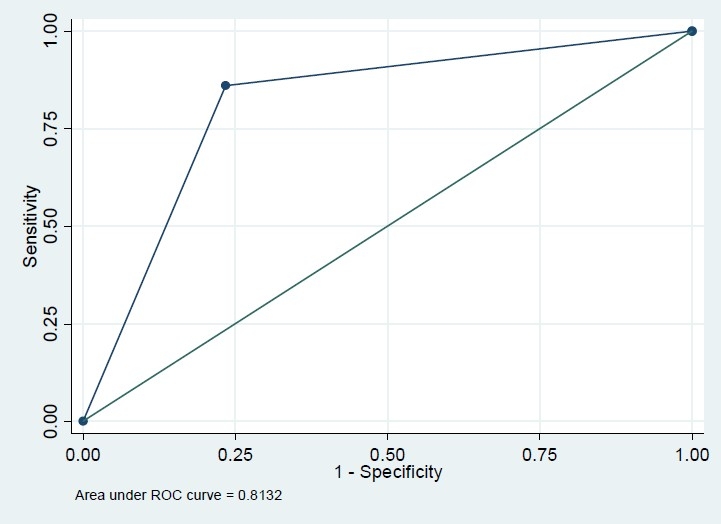
Roc plot for FBS>126 (reference variable) and HbA1c>6 (classification variable).

**Figure 2:**
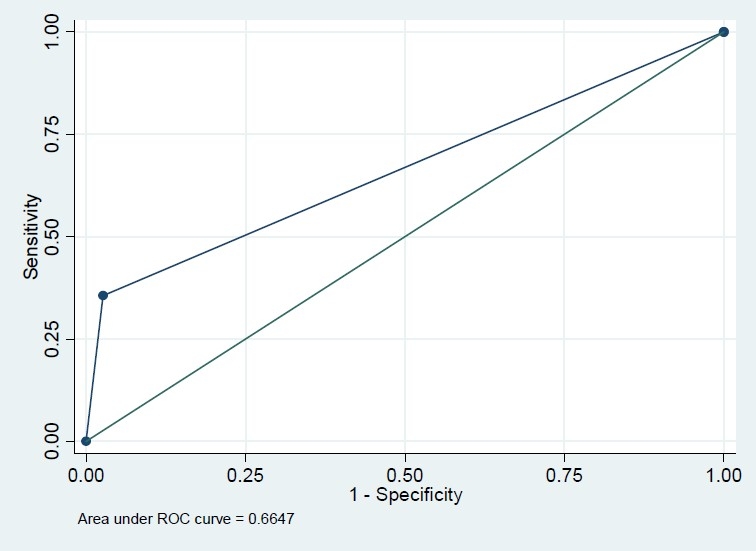
Roc plot for HbA1c>6 (reference variable) and FBS>126 (classification variable).

**Figure 3:**
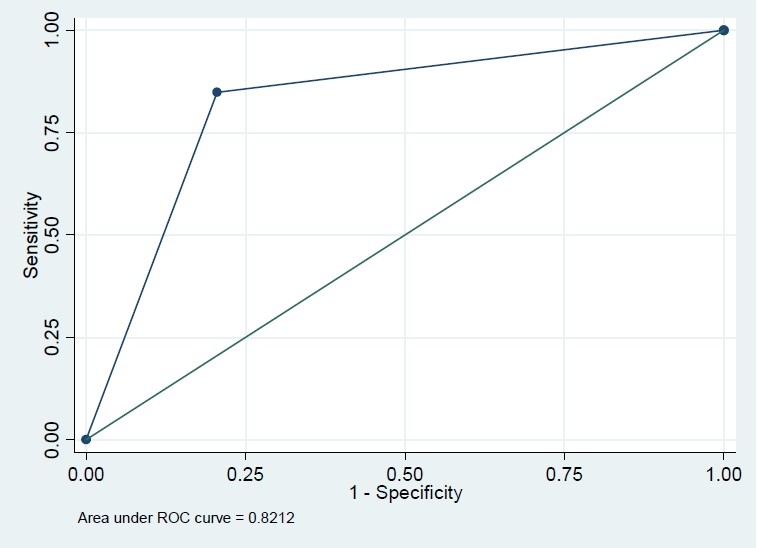
Roc plot for FBS >126 (reference variable) and HbA1c>6.15 (classification variable).

**Figure 4:**
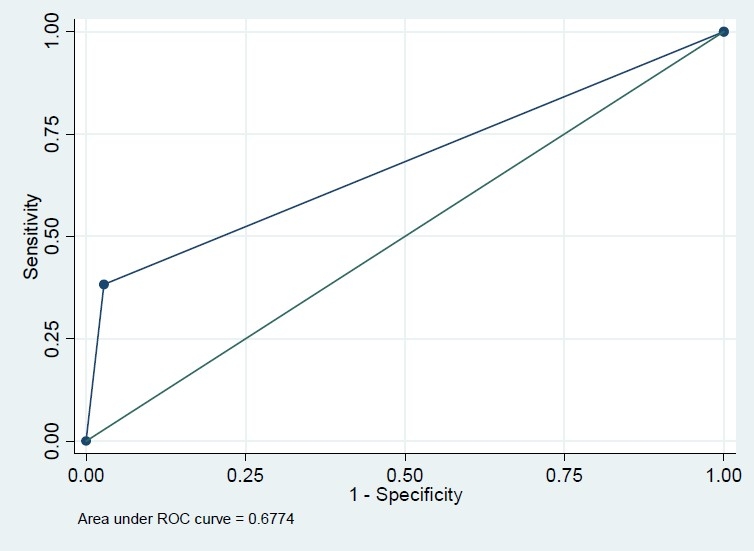
Roc plot for HbA1c>6.15 (reference variable) and FBS>126 (classification variable).

Among 604 recruited subjects, more than 60% were female but this percent was comparable in diabetics and none-diabetics (56.3% versus 64.6%, P=0.171). The mean age in diabetics was significantly greater than that in non-diabetics (43.8 versus 52.9 years, P<0.001). The difference of BMI in diabetics and non-diabetics was also significant (24.5 versus 26.2, P<0.001) (Table 1). The Pearson correlation coefficient of FBS and HbA1c was 0.74 (P<.001); however, this correlation coefficient was significantly greater in those who had FBS>126 mg/dl (r=0.73, n=79 versus r=0.23, n=525, P<0.001). Based on this finding, we modeled the association of these two variables and also their sensitivity and specificity classified by FBS because of different patterns of association in diabetic and non-diabetic subjects. In univariate analysis, the association of FBS and HbA1c with variables sex, BMI and age were significant (P<0.001) in just non-diabetics. However, they were not significant in multivariate analysis. The crude and adjusted regression coefficients of FBS in the prediction of HbA1c were greater in FBS>126 mg/dl subgroup compared with those who had normal FBS (crude coefficients: 0.18 versus 0.13, adjusted coefficients: 0.18 versus 0.09) which means that FBS could predict HbA1c in diabetics more precisely (Table 2). Similarly, the crude and adjusted regression coefficients of HbA1c in the prediction of FBS were greater in FBS>126 mg/dl subgroup compared with those who had normal FBS (crude coefficients: 2.91 versus 0.38, adjusted coefficients: 2.89 versus 0.27) which means that HbA1c could predict FBS in diabetics more precisely (Table 2). Based on conventional cutoff point of 6%, the sensitivity, specificity, positive predictive value and negative predictive value of HbA1c in the prediction of FBS>126 mg/dl were 86%, 78%, 36%, and 97%, respectively. HbA1c>6 increased the chance of diabetics almost by 23% (pre-test=13% to post-test=36%), while HbA1c≤6 increased the chance of non-diabetics by 10% (from 87% to 97%). Our ROC curve showed that 6.15% is the best cutoff point graph (Figures 1–4). Based on this optimum cutoff point, the sensitivity, specificity, positive predictive value and negative predictive value of HbA1c in the prediction of FBS>126 mg/dl were 85%, 79%, 38%, and 97%, respectively. HbA1c>6 increased the chance of diabetes by around 25% (from 13% to 38%), while HbA1c≤6 increased the chance of non-diabetics by 10% (from 87% to 97%, Table 3). On the other hand, the accuracy of FBS>126 mg/dl in the prediction of high HbA1c based on these two cutoff points of 6% and 6.15% was comparable (54% versus 56%). Although the specificity of FBS for HbA1c>6 and HbA1c>6.15 was exactly the same (97%), the sensitivity of the latter cut point was slightly greater (38% versus 36%, Table 3).

**Table 1:** Summary statistics of subjects classified in diabetic and non-diabetic subgroups based on FBS >126 mg/dl.

Categorical Variable		Non-diabetics	Diabetics	Univariate P-value
		Frequency	(%) Frequency (%)	
Sex	Female	296 (56.3)	51 (64.6)	0.171
	male	229 (43.7)	28 (35.4)	
Marriage status	Married	456 (86.9)	77 (97.47)	٠.006
	single	69 (13.1)	2 (2.53)	
Education	Illiterate	35 (6.7)	11 (13.92)	٠.072
	Primary	164 (31.2)	25 (31.65)	
	Diploma	226 (43)	34 (43.04)	
	Academic	100 (19.1)	9 (11.39)	
Jobs	No job	49 (9.3)	6 (7.59)	0.102
	Self employee	116 (22.1)	13 (16.46)	
	Employee	147 (28)	17 (21.52)	
	Housekeeper	204 (38.8)	43 (54.43)	
	Student	9 (1.7)	--	
Self-reported diabetes	Yes	30 (5.71)	60 (75.95)	<0.001
	No	495 (94.29)	19 (24.05)	
Family history of diabetes	Yes	129 (24.57)	33 (41.77)	0.001
	No	308 (58.67)	29 (36.71)	
	Don’t know	88 (16.76)	17 (21.52)	
Numeric variables		Mean (SD)	Mean (SD)	
Age		43.8 (14.6)	52.9 (11.3)	<0.001
BMI		24.5 (3.8)	26.2 (4.08)	<0.001
FBS		91.2 (12.4)	200.4 (77.3)	<0.001
A1c		5.61 (0.73)	7.88 (1.92)	<0.001

**Table 2:** Prediction of HbA1c based on FBS, and the prediction of FBS based on HbA1c.

FBS≤126 mg/dl				FBS>126 mg/dl			
**Crude**		Adjusted*		**Crude**		Adjusted*	
Beta	P-value	Beta	P-value	Beta	P-value	Beta	P-value
**Prediction of HbA1c based on FBS**							
0.13	<0.001	0.09	<0.001	0.18	<0.001	0.18	<0.001
**Prediction of FBS based on HbA1c**							
0.38	<0.001	0.27	<0.001	2.91	<0.001	2.89	<0.001

* The adjusted values were computed in a multivariable regression model with sex, BMI and age as independent variables.

**Table 3:** Comparison of sensitivity and specificity achieved for the diagnosis of diabetes based on HbA1c, at various levels of FBS at various levels of HbA1c.

Variable	HbA1c>6 and FBS>126 mg/dl		HbA1c>6.15 and FBS>126 mg/dl	
	Gold standard FBS>126 mg/dl	Gold standard HbA1c>6%	Gold standard FBS>126 mg/dl	Gold standard HbA1c>6.15%
Sensitivity (%)	86	36	85	38
Specificity (%)	77	97	79	97
Accuracy (%)	78	90	80	80
Positive predictive value (%)	36	86	38	85
Difference between the chance of disease after the test and the chance of disease before the test (%)	23	54	25	56
Negative predictive value (%)	97	77	97	79
Difference between the chance of nondisease after the test and the chance of non-disease before the test (%)	10	9	10	8
Area Under ROC Curve (AUC) (%)	81	66	82	67

## DISCUSSION

Our results showed that the association of HbA1c with FBS was relatively strong particularly in diabetic subjects. Generally, FBS was a more accurate predictor for HbA1c compared with HbA1c as a predictor of FBS. Although the optimum cutoff point of HbA1c was >6.15%, its precision was comparable with the conventional cutoff point of HbA1c >6%. Despite the fact that previous studies highlighted a straight positive association between HbA1c and FBS,^8^–^10^ this association had not been checked in diabetic and non diabetic subgroups separately, possibly due to the study design which only included diabetics. However, Derakhshan did not found any association between HbA1c and blood glucose.^11^ This could be explained by younger age of samples in that study and its low power (sample size=50). In a similar study on diabetics and people with impaired blood glucose, the sensitivity and the specificity of HbA1c as a diagnostic test were 88% and 93.75%, respectively.^29^ This is approximately similar to our findings for sensitivity and slightly different for specificity which might be explained by the difference between sample populations. HbA1c had greater sensitivity and negative predictive value in detection of FBS>126 mg/dl. Hence, a low HbA1c is a strong evidence to rule out diabetes. Although the specificity and positive predictive value of HbA1c were acceptable, they were not promising; thus, to confirm diagnosis, we can not only rely on a slight elevation of HbA1c. This issue has been emphasized in other studies as well.^17^ Therefore, we have explored alternative explanations such as anemia or abnormal hemoglobin which may generate false positive results. The limitation of using HbA1c as a screening test has been discussed explicitly;^13^ apart from drugs and anemia, it was mentioned that the standardization of tests and using different assays are other limitations in using HbA1c for screening. However, these are not the cases in our study due to the fact that we used the same laboratory method and laboratory centre for all subjects. Moreover, the higher cost of HbA1c could be another limitation in recommendation of HbA1c as a screening test. Regarding the cutoff points, although the optimum cutoff point (6.15%) had an AUC equal to 89%, the estimated parameters including the sensitivity and specificity of the test were similar to the conventional cutoff point of 6%. Optimum cutoff points varied in different studies. For example, in Gomyo study, optimum cutoff point was estimated 5.5^17^ and Rohlfing found a cutoff point of 6.2.^8^ The source of this variation might be rooted in demographic characteristics of population such as race or gender,^14^,^15^ and also in their lab techniques. Furthermore, diabetes control which is indicated by HbA1c might be affected by age distribution, and it is better controlled in younger ones.^30^ Therefore, this variation might be explained with age distribution of sample. Although the study was performed in Kerman city, we might extrapolate our findings to the urban citizens since the ethnicity and demographic of Kermanian people are more or less comparable with other parts of Iran. However, we acknowledge the limitation of generalization due to differences in life styles in different cities. In addition, as another limitation in our study, we did not use glucose tolerance test or multiple FBS tests, which have more accuracy, to define diabetes; since we got blood samples from general population, any complicated test could increase the percentage of missing data therefore we relied on the results of a single FBS. In conclusion, FBS sounds more reliable to separate diabetics from non-diabetics. However, in any case one would like to use HbA1c in screening, the conventional cutoff points of 6% is an acceptable threshold for discrimination of diabetics and non-diabetics, although 6.15% increased slightly the overall accuracy.
